# *Parkin* gene mutations are not common, but its epigenetic inactivation is a frequent event and predicts poor survival in advanced breast cancer patients

**DOI:** 10.1186/s12885-019-6013-6

**Published:** 2019-08-20

**Authors:** Khushnuma Wahabi, Ahmad Perwez, Shabeena Kamarudheen, Zafar Iqbal Bhat, Anurag Mehta, M. Moshahid A. Rizvi

**Affiliations:** 1 Genome Biology Laboratory, Department of Biosciences, Jamia Millia Islamia, New Delhi, 110025 India; 2 Department of Laboratory & Transfusion Services and Director Research, Rajiv Gandhi Cancer Institute, Rohini, Delhi 110085 India

**Keywords:** PARK-2 gene, Mutation, Methylation, Expression, Breast cancer

## Abstract

**Background:**

Progression of breast cancer involves both genetic and epigenetic factors. *Parkin* gene has been identified as a tumor suppressor gene in the pathogenesis of various cancers. Nevertheless, the putative role of *Parkin* in breast cancer remains largely unknown. Therefore, we evaluated the regulation of *Parkin* through both genetic and epigenetic mechanisms in breast carcinoma.

**Method:**

A total of 156 breast carcinoma and their normal adjacent tissue samples were included for mutational analysis through SSCP, and sequencing. MS-PCR was employed for methylation study whereas *Parkin* protein expression was evaluated using immunohistochemistry and western blotting. For the survival analysis, Kaplan–Meier curve and Cox’s proportional hazard model were used.

**Results:**

In expression analysis, *Parkin* protein expression was found to be absent in 68% cases of breast cancer. We found that aberrant promoter methylation of *Parkin* gene is a frequent incident in breast cancer tumors and cell lines. Our MS-PCR result showed that *Parkin* promoter methylation has a significant role (*p* = 0.0001) in reducing the expression of *Parkin* protein. Consistently, expression of *Parkin* was rectified by treatment with 5-aza-2-deoxycytidine. We also found significant associations of both *Parkin* negative expression and *Parkin* promoter methylation with the clinical variables. Furthermore, we found a very low frequency (5.7%) of *Parkin* mutation with no clinical significance. In survival analysis, patients having *Parkin* methylation and *Parkin* loss had a worse outcome compared to those harboring none of these events.

**Conclusion:**

Overall, these results suggested that promoter methylation-mediated loss of *Parkin* expression could be used as a prognostic marker for the survival of breast cancer.

**Electronic supplementary material:**

The online version of this article (10.1186/s12885-019-6013-6) contains supplementary material, which is available to authorized users.

## Background

Globally, breast cancer is the most fatal malignancy in women and a second major cause of cancer-related deaths among females [1]. In India, the incidence of breast cancer cases has overtaken cervical cancer as the most commonly diagnosed cancer among women, witnessing a rapid rise and more likely to increase in the future [2].

*Parkin* (PARK2 or PRKN) gene which spans more than 1.38 Mb is one of the largest human genes maps to chromosome 6q25.2-q27 [3]. *Parkin* gene lies within FRA6E region, the third most fragile site which is prone to rearrangement and breakage in tumors [3]. Alterations in *Parkin,* an E3 ubiquitin-protein ligase are mainly associated with Parkinson’s disease [4]. Nevertheless, accumulating pieces of evidence have highlighted its tumor-suppressive role in addition to the one-sided view of its ubiquitin ligase activity [4, 5]. Several studies have demonstrated *Parkin* gene alterations in a wide array of cancers including brain, breast, liver, pancreas, kidney, ovarian, cervical, and colorectal cancer [6–12]. Numerous groups have reported lack of *Parkin* expression due to mutation and hypermethylation in a variety of cancers [8, 13–15]. Besides, down-regulation and copy number loss of the *Parkin* are common events in pancreatic cancers [11]. Moreover, *Parkin* is found to regulate energy metabolism namely Warburg effect thereby suppressing tumorigenesis [16]. Recently, *Parkin* has been suggested as a key player involved in different hallmarks of cancer cell [17]. Amazingly, a functional interplay has been reported between the *Parkin* and p53, a well-established tumor suppressor [16, 18]. Whereas another study indicated the role of *Parkin* in the metastasis through interaction with HIF-1α (hypoxia-inducible factor 1α) thus highlighted the pivotal role of *Parkin* in tumor suppression [19]. Collectively, the aforementioned studies emphasized that downregulation of *Parkin* may promote cancer however the precise mechanism of *Parkin* inactivation remains unexplored mainly in breast cancer.

Aberrant promoter methylation is a widespread mechanism in cancer. It is an emerging molecular marker which raises the hopes for the development of novel therapeutics in combating cancer [20, 21]. Recent studies have reported aberrant methylation at *Parkin* promoter among acute lymphoid leukemia (ALL), chronic granulocytic leukemia (CGL) [15], nasopharyngeal carcinoma [22] and cervical cancer [9]. Although the precise genetic and epigenetic mechanisms contributing to *Parkin* loss in breast cancer remain elusive, this prompted us to investigate the possible mechanisms as well as the potential role of *Parkin* gene in breast cancer.

## Methods

### Ethical approval

The present study was approved by the Ethics Committee and Institutional Review Board (ECIRB) of Jamia Millia Islamia, New Delhi and Rajiv Gandhi Cancer Institute and Research Centre, New Delhi, India. Each participating patient signed informed written consent.

### Tumor specimens and cell lines

The study comprised of 156 pairs of histologically confirmed breast carcinoma and their adjacent normal tissue samples (without any tumor cell infiltration) from sporadic breast cancer patients undergoing biopsies at Rajiv Gandhi Cancer Research Institute and Research Centre, New Delhi, from 2013 to 2017. After surgery, all samples were instantly put in liquid nitrogen and kept in − 80 °C until further use. Clinicopathological parameters of the breast cancer patients were obtained from the hospital database (Additional file 1: Table S1). The exclusion criteria for the study were metastasized cases from other organs, cases having a prior history of any cancer and prior exposure to chemotherapy and radiation. Three breast cancer cell lines; MCF-7, MDA-MB-231, MDA-MB-468, and one normal HEK-293 (Human embryonic kidney) cells were procured from National Centre for Cell Sciences (NCCS) Pune, India. The cells were grown as a monolayer culture in Dulbecco’s modified Eagle’s medium containing 10% fetal bovine serum (Gibco, Thermoscientific, South American origin) and antibiotics (100 U penicillin and, 100 mg L^−1^streptomycin) at 37 °C in a humidified atmosphere of 5% CO_2_ and were subcultured twice a week [23]. Stock culture of the cell lines was maintained in the exponential growth phase by passaging as monolayer culture, the dislodged cells were suspended and reseeded routinely in complete medium.

### Nucleic acid extraction

Genomic DNA was extracted using proteinase K/phenol-chloroform protocol from a total of 156 breast cancer (confirmed by a pathologist) and adjacent normal tissues, and also from four cell lines (MCF-7, MDA-MB-231, MDA-MB-468, and HEK-293). Besides, a total RNA was isolated by TRIzol Reagent from cell lines (Invitrogen) according to the manufacturer’s instructions.

### PCR–SSCP and sequencing

A total of 12 exons were amplified to reveal any somatic mutation of *Parkin* by single-stranded conformational polymorphism (SSCP). Extracted DNA was used for PCR amplification using primers (Additional file 1: Table S2) and amplification conditions as described earlier [9]. The amplified products were visualized by electrophoresis using 2% agarose gel and stained with ethidium bromide. SSCP protocol was followed as mentioned earlier [9]. Samples that demonstrated differences in band-shifts with respect to the wild-type bands were categorized as mutants. To confirm mutations those samples were re-amplified in 40 μL reactions for DNA sequencing using forward and reverse primers. For each sample sequencing was repeated to minimize sequencing artifacts and to confirm mutations. The BLAST tool was employed for pair-wise nucleotide sequence alignment.

### TCGA (the Cancer genome atlas) and COSMIC (catalogue of somatic mutations in Cancer) analysis

To analyze the *Parkin* genetic alterations among breast tumors in TCGA (The Cancer Genome Atlas) we used the cBioPortal database (available at www.cbioportal.org). TCGA (http://cancergenome.nih.gov/) is a publically available reservoir containing 33 types of cancer having more than eleven thousand human tumors with their clinical and molecular phenotypes [24, 25]. The COSMIC (Catalog of Somatic Mutations in Cancer) database (https://cancer.sanger.ac.uk/cosmic) analysis was done to figure out mutations of *Parkin* (PARK2). Pie charts showing distribution and substitutions of *Parkin* mutations in breast cancer were obtained.

### Oncomine database and UALCAN analysis

Oncomine database was exploited to investigate the mRNA expression of *Parkin* in breast cancer using the criteria of *p*-value less than 10^–4^ and fold change more than 1.5 [26]. The top 10% of the resulted lists were examined (https://www.oncomine.org/). The *Parkin* mRNA expression was analyzed in TCGA breast and Compendia cell lines datasets. Moreover, UALCAN tool (http://ualcan.path.uab.edu/index.html) was also used to correlate the clinicopathological parameters among breast cancer in TCGA data.

### Bisulphite-modification and MSP (methylation specific polymerase chain reaction)

**Fig. 1:**
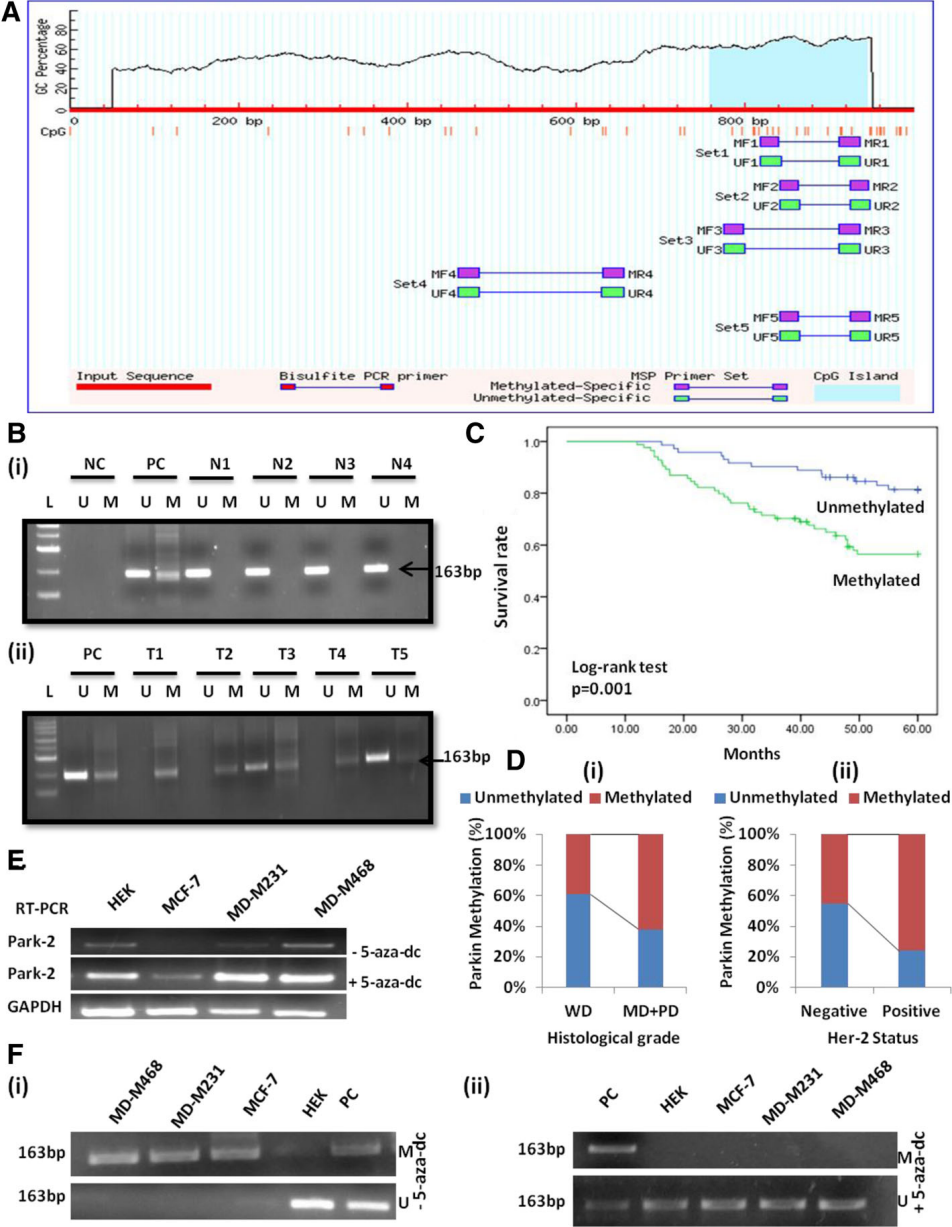
*Parkin* (PARK-2) methylation analysis. **a** Graphical representation of CpG islands (187) in the *Parkin* promoter region taken from MethPrimer (https://www.urogene.org/methprimer/); Criteria used: Island size > 100, GC Percent > 50.0, Obs/Exp > 0.60 **b** MS-PCR gel pictures, representing methylation of *Parkin* in **(i)** normal breast tissues & **(ii)** breast carcinoma tissues, PC-Positive control, NC-Negative control & L- Ladder (100 bp). **c** The survival curve was analyzed according to promoter methylation of Parkin protein (SPSS version 17.0). **d** Frequency distribution of *Parkin* methylation in **(i)** Histological grade **(ii)** Her-2 status (*p* < 0.05). Demethylating treatment with 5-aza-dC restored *Parkin* expression and unmethylated status in breast cancer cell lines. **e** *Parkin* mRNA expression through RT-PCR, showing that demethylating treatment with 5-aza-dC restored *Parkin* expression in MCF-7, MDA-MB-231, and MDA-MB-468 cell lines. HEK a non-tumor derived cell line was used as a positive control. GAPDH was amplified as an internal control. **f** Methylation-specific PCR of *Parkin* promoter in breast cancer cell lines MCF-7, MD-M231 & MD-M468 before **(i)** and after **(ii)** 5-aza-dC treatment. HEK was used as a positive control. PC- Positive control totally methylated and unmethylated bisulfite converted human genomic DNA

DNA isolated from the tissues was employed in bisulfite modifications by using EZ DNA Methylation-Gold TM kit (Zymo Research, USA) according to manufacturer’s instruction. In *Parkin* promoter region one CpG island (187 bp) was found just before the transcription site through the Methprimer tool (https://www.urogene.org/methprimer/) (Fig. 1a). MSP reaction was carried out using the unmethylated and methylated primers in a final volume of 25 μL (Additional file 1: Table S2). PCR reaction conditions were, an initial denaturation at 95 °C for 5 min, after that 40 cycles of denaturation at 95 °C for 45 s, followed by annealing step at 63 °C for 35 s, and extension was done at 72 °C for 45 s, a last terminating cycle of final extension was carried out at 72 °C for 7 min. Commercially available methylated and unmethylated bisulfite converted human genomic DNA (Zymo Research Corp., Orange, CA) were used as positive controls of methylated and unmethylated alleles. As a negative control double distilled water (ddH2O) was used in each PCR reaction. Amplified PCR products were then visualized on 2% agarose gels with 100 bp DNA ladder as a standard reference and photographed using Gel Doc (Bio-Rad laboratories, CA, USA) under UV (ultraviolet) illumination.

### 5-Aza-2-deoxycytidine treatment

Three breast cancer (i.e. MCF-7, MDA-MB-231, and MD-MB-468) and one non-tumor derived HEK cell lines were seeded at a density of 2 × 10^5^ cells into six-well plates. Next day, Iscove’s modified Dulbecco’s medium containing 10 μmol/L 5-aza-dC (Sigma, USA) was added and changed every 24 h. After 96 h, RNA and DNA were isolated for RT-PCR and MSP analysis respectively.

### RT-PCR (reverse transcriptase PCR)

RNA extracted from the cell lines were then used for the synthesis of complementary DNA (cDNA) using iScript™ Reverse Transcription Reagents (Bio-Rad Laboratories, Inc.) and were stored at − 80 °C. PCR reaction was carried out in a final volume of 25 μl, using 2 μl of cDNA, 1 U AmpliTaq Gold DNA Polymerase (Applied Biosystems, Foster City, CA) 0.2 mM dNTPs, 1.5 mM MgCl2, and 20 pmol of primers as listed in Additional file 1: Table S2. PCR conditions were, an initiation of 94 °C for 10 min, followed by 35 cycles of denaturation at 94 °C for 1 min, annealing at 58 °C for 45 s and 7 min extension at 72 °C followed by a final extension of 10 min at 72 °C. Additionally, in each reaction, a set of primers specific for the *GAPDH* gene (Applied Biosystems) was included as an internal control. Amplified aliquots were then visualized on 2.0% agarose gels. For semi-quantitative analysis, Quantity One v 4.4.0 software (Bio-Rad, USA) was used.

### Immunohistochemistry (IHC)

Immunohistochemical staining was carried out on formalin-fixed paraffin-embedded tissue blocks of each sample. The 3–4 μm thin tissue sections were then taken on Poly-L-lysine coated slides. The protocol was followed as described previously [9]. The slides were incubated in a humidified chamber with 1:100 dilution of anti-*PARK-2* antibody (cat #ab15954, Abcam) at 4 °C for 24 h. After washing with PBS thrice, slides were next incubated with biotinylated secondary antibody and with an avidin-horseradish peroxidase for 25–30 min. To visualize antigen-antibody reaction 3, 3′-diaminobenzidine (DAB) substrate (DAB substrate kit, Vector Laboratories) was added followed by a counterstaining with hematoxylin dye. Normal adjacent breast tissues were used as positive controls.

### Staining interpretation

Stained slides were evaluated by two expert histopathologists at 100X and 400X magnifications under the light microscope. At least three tissue cores from each case were evaluated. The staining < 5% was considered as negative expression and more than 5% were measured as positive.

### Western blotting

Protein was extracted from the breast cancer cell lines and tissues with a RIPA lysis buffer (150 mM NaCl, 1% sodium deoxycholate, 1% Triton X-100, 0.1% SDS, 50mMTris_HCl, pH 7.5/2mMEDTA) and was quantified by using the BCA kit (Pierce). Samples of 40 μg of protein were loaded per well in SDS-PAGE followed by transfer onto nitrocellulose membranes (Bio-Rad). Non-specific binding was blocked using 5% bovine serum albumin (BSA) containing 0.05% Tween-20 for 1 h. Subsequently, incubation with primary antibodies, anti-*PARK-2* (1:1000; cat #ab15954, Abcam) and *GAPDH* (1:1000 dilution; Santa Cruz Biotechnology) was done at 4 °C for overnight. Following washing with PBST (phosphate-buffered saline with Tween 20), membranes were re-incubated with secondary antibody conjugated with horseradish peroxidase (HRP) anti-rabbit for *PARK-2* and anti-mouse for GAPDH for 2 h at room temperature. The bands were then developed in the darkroom on photographic films through luminol method [27]. The Western blots were quantified by densitometry analysis using ImageJ software 1.46r version.

### Statistical analysis

All Statistical evaluations of data were done through the SPSS (Statistical Package for the Social Sciences), version 17.0 for the window. Fisher’s exact test was used for all the comparisons to evaluate the statistical significance with *P* values < 0.05 and the confidence intervals were quoted at 95% level. Overall survival (OS) was analyzed from the date of surgery to date of the event. Besides, Univariate analysis of time to death (as a result of cancer) was done using the Kaplan-Meier method, and the log-rank test was used to compare the survival times. Univariate and multivariate analysis of the prognostic factors were examined by Cox’s proportional hazard model to identify independent variables predictive of OS. The P value < 0.05 was considered statistically significant for all methods.

## Results

### Mutational analysis of *Parkin* gene

**Fig. 2:**
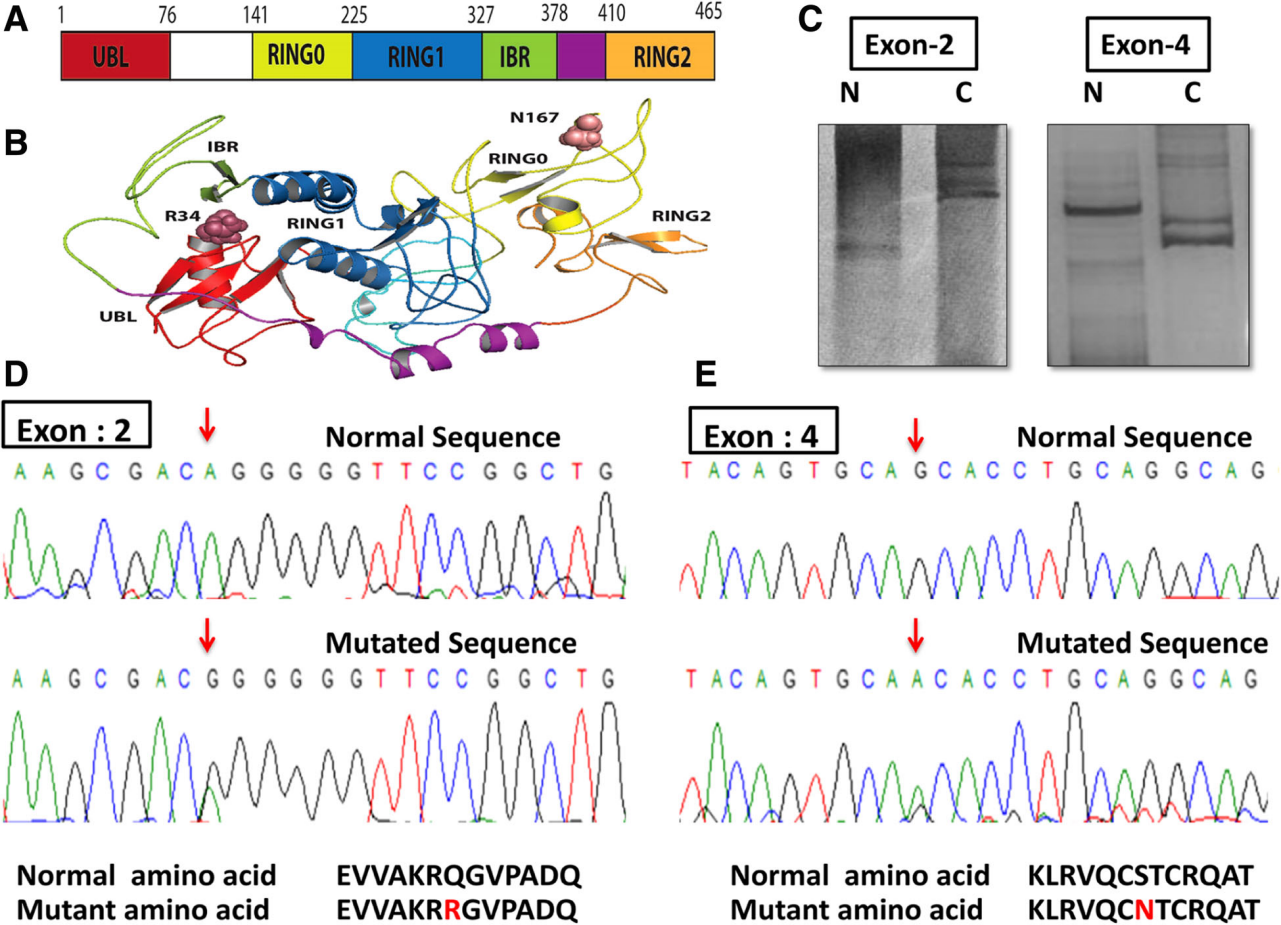
Representation of *Parkin* (PARK-2) somatic mutations. **a** Illustration of *Parkin* 2D structure showing different domains (**b**) a ribbon representation of the *Parkin* conformation highlighting somatically mutated residues taken from PyMOL (version 1.7.4.5 Edu.) **c** Gel pictures of SSCP showing band shift, N-Normal Adjacent tissue, C-Cancerous tissue. **d** Sequencing histogram of Exon 2 with transition A → G at nucleotide position 235 leading to the conversion of glutamine (Q) to arginine (R) at codon 34 & (**e**) Exon 4 showing transition G → A at nucleotide position 634 leading to the conversion of Serine (S) to asparagine (N) at codon 167

In the mutational analysis, we found that only 5.7% (9/156) breast cancer cases have somatic mutations in exon 2 and exon 4 of *Parkin* gene (Fig. 2d & e), the same were absent in the normal adjacent tissues. Out of these nine cases, six samples demonstrated A to G transition at nucleotide position 235 leading to conversion of glutamine to arginine at codon 34 in exon 2, while other three samples showed the transition of G to A at nucleotide position 634 leading to conversion of serine to asparagine at codon 167 in exon 4 (Additional file 1: Table S3, Fig. 2a-e). We for the first time reported these two novel somatic mutations; Glu34Arg and Ser167Asp, which were not found in the available list of COSMIC (Additional file 2: Figure S2). Markedly, all mutated cases coincided with the loss of *Parkin* protein, although we did not find any significant association of these mutations (*p* > 0.05). Our result is consistent with the TCGA data (cBioPortal: http://www.cbioportal.org/) and COSMIC databases showing that the mutation frequency of *Parkin* is very low (2.3%) in breast cancer (Additional file 2: Figures S1, S2 and S3).

### *Parkin* protein expression is frequently absent in breast tumors

**Fig. 3:**
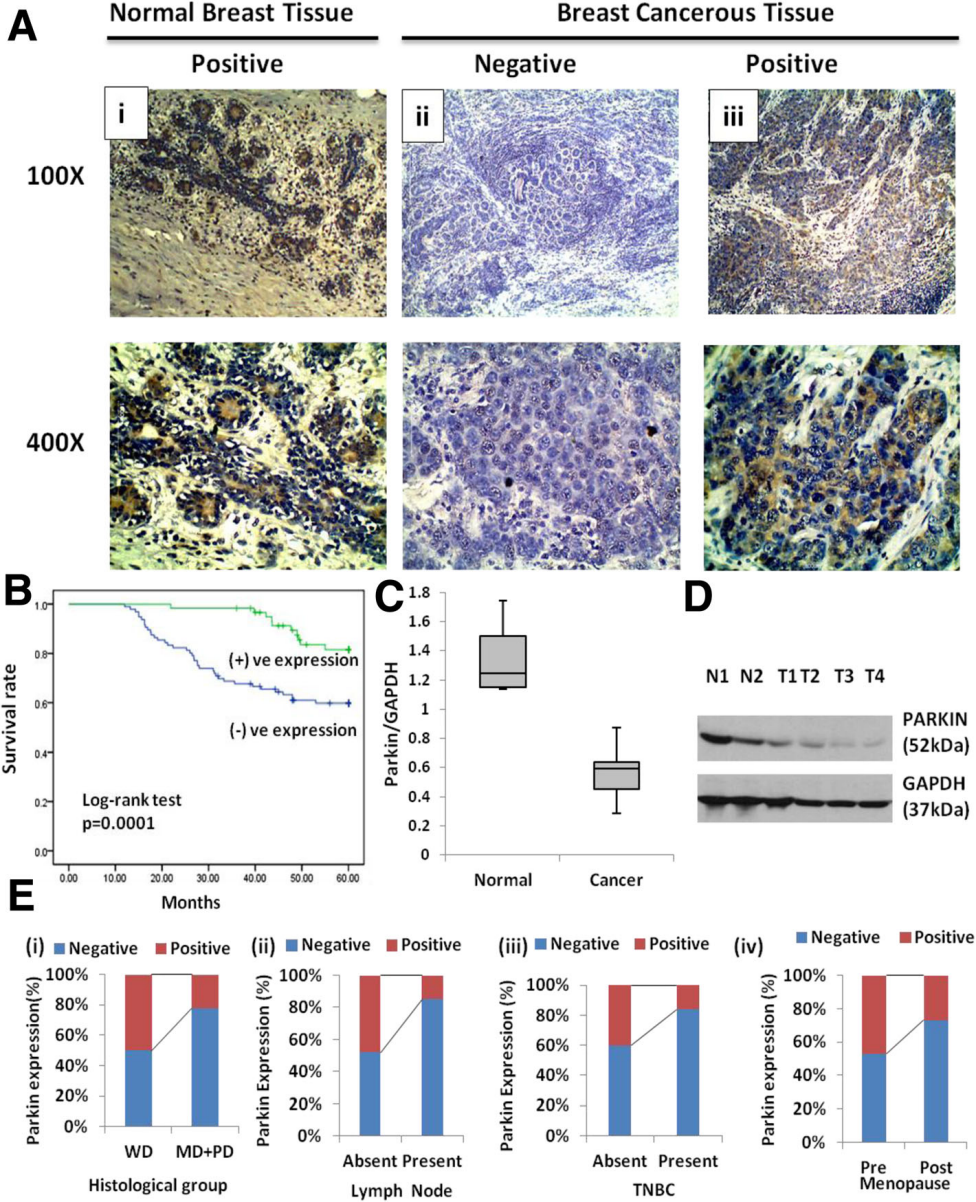
*Parkin* (PARK-2) expressional analysis. **a** Immunohistochemical analysis of *Parkin* protein in breast tissue at 100X and 400X magnifications. **(i)** *Parkin* positive expression in normal tissue, **(ii)** *Parkin* negative expression, **(iii)** *Parkin* positive expression in breast carcinoma respectively (Scale bar: 1000 μm). **b** The Survival curve was analyzed according to the expression status of *Parkin* protein (SPSS version 17.0). **c** *Parkin* protein expression in breast cancer tissues, N-Normal, T-Tumor. **d** Frequency distribution of *Parkin* expression with **(i)** Lymph node, **(ii)** histological grade and **(iii)** TNBC cases (p < 0.05). **e** Distribution of the *Parkin*/*GAPDH* ratio in normal and breast cancer tissues

**Fig. 4:**
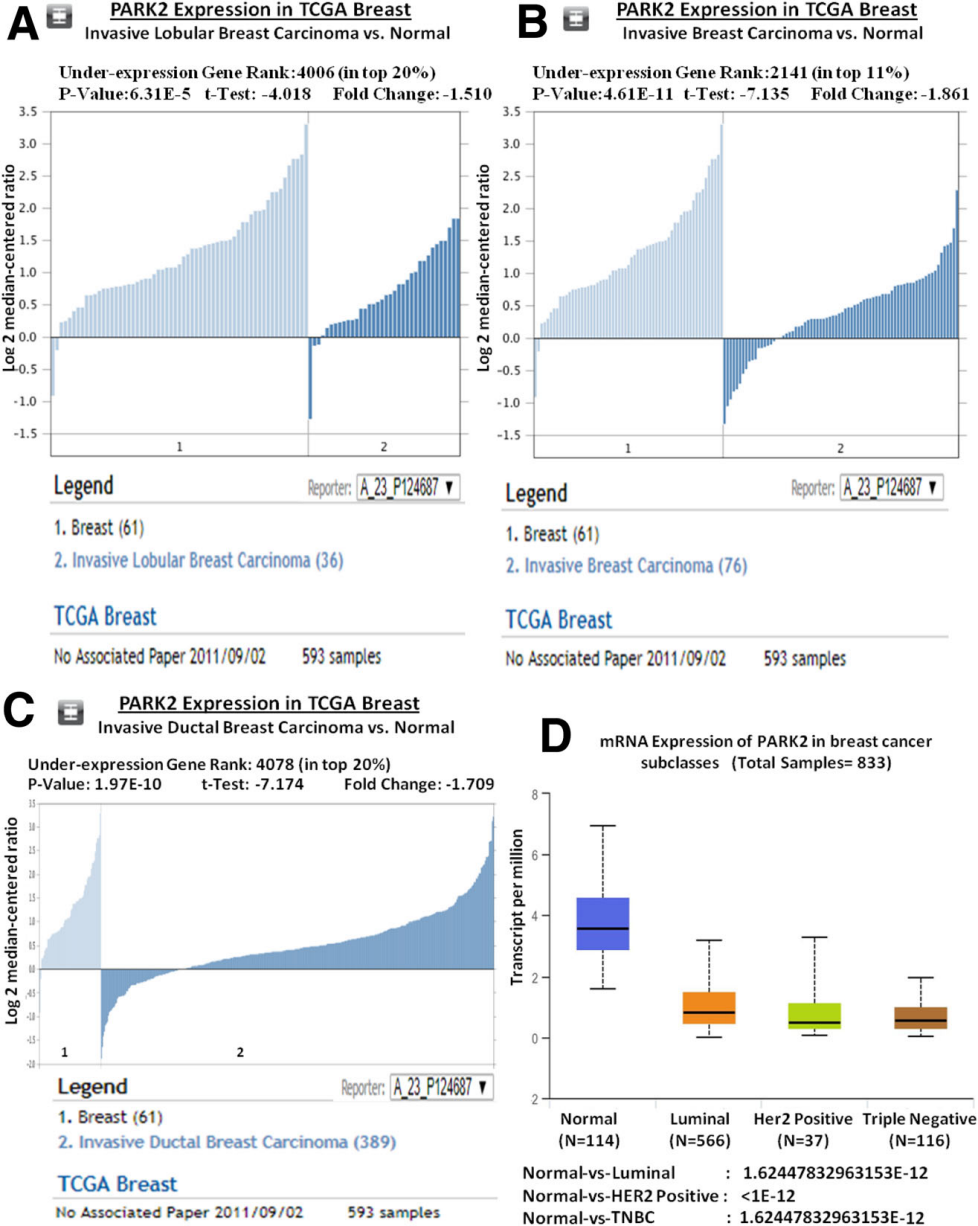
Oncomine analysis showing loss of *Parkin* (PARK-2) expression in different types of breast carcinoma in TCGA. **a** Invasive lobular breast carcinoma vs. normal tissues, **b** Invasive breast carcinoma vs. normal tissues, and **c** Invasive ductal breast carcinoma vs. normal tissues using TCGA breast data. **d** Relation of *Parkin* mRNA expression among different subgroups of breast cancer such as Normal, Luminal, Her2 Positive and Triple-negative breast cancers. Oncomine database (https://www.oncomine.org/); TCGA: The Cancer Genome Atlas (Significant value: *P* < 0.05)

Immunohistochemical study showed predominant cytoplasmic expression of *Parkin* protein in normal breast tissues (Fig. 3a). The IHC results revealed *Parkin* protein to be frequently absent in 68% (106/156) cases (Fig. 3a (i), (ii) & (iii)). Interestingly, results of western blot were found to be very well corroborated for the *Parkin* protein expression where we also found a lower level of *Parkin* protein in cancer tissues in contrast to normal tissues (Fig. 3c & d). Oncomine study demonstrated that expression of *Parkin* mRNA is low in different breast cancer types in comparison to normal tissues (Fig. 4a-c). The Compendia cell lines dataset also showed lower expression of *Parkin* mRNA in breast cancer cell lines in comparison to most of the other cancer cell lines (Additional file 2: Figure S4).

### Hypermethylation of *Parkin* in breast cancer tumors

Promoter methylation of *Parkin* was detected in 54% (84/156) cases of breast tumors (Fig. 1b; (i) and (ii)). Interestingly, we found a strong correlation between the *Parkin* methylation and *Parkin* loss (*p* = 0.0001). The results showed that methylation frequency coincided with the lower expression of *Parkin* protein, as evident in 82% (69/84) cases (Table 1).

**Table 1:** Correlation of *Parkin* promoter methylation with *Parkin* protein expression

Parkin Promoter	Parkin expression^a^		Total (%)	*p* Value^b^	OR (95%CI)
	Negative	Positive			
Unmethylated	37 (51)	35 (49)	72 (46)	0.0001*	0.2298 (0.1113–0.4744)
Methylated	69 (82)	15 (18)	84 (54)		
Total (%)	106 (68)	50 (32)	156 (100)		

^a^Protein expression through IHC (Immunohistochemistry)

^b^Fisher’s Exact test, *Significant Correlation (*P* < 0.05)

### *Parkin* promoter methylation and loss of *Parkin* expression

To verify the role of *Parkin* promoter methylation in the loss of *Parkin* protein expression, the mRNA level of *Parkin* was checked in three breast cancer cell lines: MCF-7, MDA-MB-231, and MDA-MB-468. The MCF-7 was found to be *Parkin* negative, while MDA-MB-231 & MDA-MB-468 have shown very low expression of *Parkin* (Fig. 1e). Additionally, these cell lines were then treated with 5-aza-dC, a demethylating agent. Following the treatment, restoration of *Parkin* mRNA expression was witnessed in MCF-7 cells while MDA-MB-231 & MDA-MB-468 cell lines showed relatively increased expression (Fig. 3e). Furthermore, to confirm the result MSP was performed and found only unmethylated bands for all the cell lines (Fig. 3f (i) & (ii)).

### Correlation of *Parkin* protein expression with clinicopathological parameters and patient survival

While finding the statistical correlation of *Parkin* protein expression with clinical parameters of the patients, we observed a significant correlation of *Parkin* negative expression with the histological grade (*p* = 0.0001), Lymph node (*p* < 0.0001), Menopause (*p* = 0.028) and TNBC status (*p* = 0.003) (Table 2) (Fig. 3e (i), (ii) & (iii)). While examining the TCGA breast dataset using UALCAN tool, we speculated that the expression of *Parkin* mRNA is significantly correlated with the TNBC cases as revealed by our data. On contrary, TCGA data also demonstrated a significant association between the *Parkin* mRNA expression and Her2 positivity which we failed to get in our study that might be due to differences in population or sample size. Furthermore, Kaplan-Meier survival curve demonstrated that *Parkin* positive expression has a better mean survival time of 58.2 months than the negative expression 46.6 months (p = 0.0001)(Fig. 3b). In a stratified univariate analysis, the prognostic value of *Parkin* expression became even more pronounced for OS along with the clinical stage, histological grade and lymph node thereby chosen as the factors to be included in the same Cox regression model. A multivariate analysis also validated that clinical stage (relative risk, 1.476; 95% CI: 1.094–1.990, *p* = 0.011), histological grade (relative risk, 3.198; 95% CI: 1.412–7.245, *p* = 0.005), lymph node (relative risk, 2.194; 95% CI: 1.150–4.186, *p* = 0.017) and *Parkin* expression (relative risk, 0.057; 95% CI: 0.008–0.418, p = 0.005) are independent prognostic factors for OS (Table 3).

**Table 2:** Correlation of *Parkin* protein expression with clinical parameters among breast cancer patients

S. No.	Variables		Total	Parkin Expression^a^		*P* value^b^	OR (95%CI)
				Positive	Negative		
1	Age (Years)	< 50	30	9 (30)	21 (70)	0.832	1.1255(0.4738–2.6737)
		≥50	126	41 (33)	85 (67)		
2	Weight (Kg)	< 60	75	26 (35)	49 (65)	0.607	0.7935 (0.4046–1.5562)
		≥60	81	24 (30)	57 (70)		
3	Tumour size (cm)	< 4’	73	29 (40)	44 (60)	0.060	0.5139 (0. 2599–1.0161)
		≥4	83	21 (25)	62 (75)		
4	Clinical Stage	I + II	88	29 (33)	59 (67)	0.863	0.9090 (0.4606–1.7941)
		III + IV	68	21 (31)	47 (69)		
5	Histological^c^ grade	WD	54	27 (50)	27 (50)	0.0001*	0.2911 (0.1435–0.5906)
		MD + PD	102	23 (22)	79 (78)		
6	Lymph Node	Negative	81	39 (48)	42 (52)	< 0.0001*	0.1851 (0.0854–0.4014)
		Positive	75	11 (15)	64 (85)		
7	Menopause	Pre	38	18 (47)	20 (53)	0.028*	0.4134 (0.1943–0.8797)
		Post	118	32 (27)	86 (73)		
8	ER^d^	Negative	81	23 (28)	58 (72)	0.391	1.9438 (0.0190–3.7081)
		Positive	75	27 (36)	48 (64)		
9	PR^e^	Negative	103	32 (31)	71 (69)	0.721	1.1411 (0.5637–2.3099)
		Positive	53	18 (34)	35 (66)		
10	Her-2^f^	Negative	111	36 (32)	75 (68)	1.000	0.9409 (0.4463–1.9835)
		Positive	45	14 (31)	31 (69)		
11	TNBC^g^	No	106	42 (40)	64 (60)	0.003*	0.2902 (0.1240–0.6793)
		Yes	50	8 (16)	42 (84)		

^a^ Protein expression through IHC (Immunohistochemistry)

^b^ Fisher’s exact test, *Significant Correlation (*P* < 0.05)

^c^ *WD* Well differentiated, *MD* Moderately differentiated, *PD* Poorly differentiated

^d^ Estrogen receptor

^e^ Progesterone receptor

^f^ human epidermal growth factor receptor 2

^g^ Triple Negative breast cancer

**Table 3:** Univariate and multivariate overall survival analysis of different prognostic variables and *Parkin* expression in breast cancer patients by cox proportional hazard model

Variables	Univariate analysis			Multivariate analysis		
	Hazard ratio	95% CI	*P* Value	Hazard ratio	95% CI	*P* Value
Age (< 50 yr. vs. ≥50 yr.)	1.535	0.688–3.422	0.295	**–**	**–**	**–**
Weight (< 60 kg vs. ≥60 kg)	0.720	0.408–1.270	0.295	**–**	**–**	**–**
Tumour size (< 4 cm vs. ≥4 cm)	1.322	0.745–2.347	0.340	**–**	**–**	**–**
Clinical Stage (I vs. II vs. III vs. IV)	1.409	1.028–1.930	0.033*	1.476	1.094–1.990	0.011*
Histological^a^ grade (WD vs. MD + PD)	3.659	1.641–8.162	0.002*	3.198	1.412–7.245	0.005*
Lymph Node (Neg. vs. Pos.)	3.427	1.812–6.483	< 0.0001*	2.194	1.150–4.186	0.017*
Menopause (Pre vs. Post)	2.184	0.979–.4.870	0.056	**–**	**–**	**–**
ER^b^ (Neg. vs. Pos.)	0.843	0.476–1.491	0.557	**–**	**–**	**–**
PR^c^ (Neg. vs. Pos.)	0.717	0.380–1.356	0.306	**–**	**–**	**–**
Her-2^d^ (Neg. vs. Pos.)	1.784	0.999–3.184	0.050*	**–**	**–**	**–**
TNBC^e^ (No vs. Yes)	0.839	0.450–1.563	0.579	**–**	**–**	**–**
Parkin (Neg. vs. Pos.)	0.035	0.005–0.253	0.001*	0.057	0.008–0.418	0.005*

^a^ *WD* Well differentiated, *MD* Moderately differentiated, *PD* Poorly differentiated

^b^ Estrogen receptor

^c^ Progesterone receptor

^d^ human epidermal growth factor receptor 2

^e^ Triple Negative breast cancer, *Significant Correlation (*P* < 0.05)

### Correlation of *Parkin* methylation with clinicopathological parameters and patient survival

In the descriptive analysis, *Parkin* promoter methylation was correlated with different clinicopathological variables of breast cancer patients. We observed a significant correlation of *Parkin* methylation with the histological grade (*p* = 0.007) and Her-2 status (*p* = 0.001) (Fig. 1d; (i) & (ii)) (Table 4). However, no other clinical parameter showed a significant correlation with *Parkin* promoter methylation (*p* > 0.05). In survival analysis, Kaplan-Meier method also illustrated that the patients with methylated *Parkin* promoter had a mean survival time of 46.7 months as compared with 55.3 months for patients with the unmethylated *Parkin* promoter (p = 0.001)(Fig. 1c). The clinical parameters; Clinical stage, histological grade, and lymph node were found significant prognostic indicators for OS in univariate analysis, thus included as the parameters in the same Cox regression model. Results of multivariate analysis further provided the evidence that *Parkin* promoter methylation (relative risk, 2.286; 95% CI: 1.190–4.389, *p* = 0.013) is an independent prognostic factor for OS (Table 5).

**Table 4:** Correlation of PARK-2 promoter methylation with clinical parameters among breast cancer patients

S No.	Variables	Total	Parkin Promoter			*P* value^a^	OR (95%CI)
			Unmethylated		Methylated		
1	Age (Years)	< 50	30	14 (47)	16 (53)	1.000	1.0259 (0.4617–2.2791)
		≥50	126	58 (46)	68 (54)		
2	Weight (Kg)	< 60	75	36 (48)	39 (52)	0.748	1.1538 (0.6143–2.1672)
		≥60	81	36 (44)	45 (56)		
3	Tumour size (cm)	< 4’	73	37 (51)	36 (49)	0.335	1.4095 (0.7487–2.6537)
		≥4	83	35 (42)	48 (58)		
4	Clinical Stage	I + II	88	36 (41)	52 (59)	0.148	0.6154 (0.3251–1.1650)
		III + IV	68	36 (53)	32 (47)		
5	Histological grade^b^	WD	54	33 (61)	21 (39)	0.007*	2.5385 (1.2894–4.9974)
		MD + PD	102	39 (38)	63 (62)		
6	Lymph Node	Negative	81	42 (52)	39 (48)	0.151	1.6154 (0.8559–3.0487)
		Positive	75	30 (46)	45 (54)		
7	Menopause	Pre	38	23 (61)	15 (39)	0.061	2.1592 (1.0235–4.5549)
		Post	118	49 (42)	69 (58)		
8	ER^c^	Negative	81	41 (51)	40 (49)	0.264	1.4548 (0.7723–2.7404)
		Positive	75	31 (41)	44 (59)		
9	PR^d^	Negative	103	49 (48)	54 (52)	0.735	1.1836 (0.6076–2.3056)
		Positive	53	23 (43)	30 (57)		
10	Her-2^e^	Negative	111	61 (55)	50 (45)	0.001*	3.7709 (1.7351–8.1925)
		Positive	45	11 (24)	34 (76)		
11	TNBC^f^	No	106	49 (46)	57 (54)	1.000	1.0092 (0.5140–1.9812)
		Yes	50	23 (46)	27 (54)		

^a^ Fisher’s exact test, *Significant Correlation (*P* < 0.05)

^b^ *WD* Well differentiated, *MD* Moderately differentiated, *PD* Poorly differentiated

^c^ Estrogen receptor

^d^ Progesterone receptor

^e^ human epidermal growth factor receptor 2

^f^ Triple negative breast cancer

**Table 5:** Univariate and multivariate overall survival analysis of different prognostic variables and *Parkin* methylation in breast cancer patients by cox proportional hazard model

Variables	Univariate analysis			Multivariate analysis		
	Hazard ratio	95% CI	*P* Value	Hazard ratio	95% CI	*P* Value
Age (< 50 yr. vs. ≥50 yr.)	1.535	0.688–3.422	0.295	**–**	**–**	**–**
Weight (< 60 kg vs. ≥60 kg)	0.720	0.408–1.270	0.295	**–**	**–**	**–**
Tumour size (< 4 cm vs. ≥4 cm)	1.322	0.745–2.347	0.340	**–**	**–**	**–**
Clinical Stage (I + II vs. III + IV)	1.409	1.028–1.930	0.033*	1.541	1.139–2.083	0.005*
Histological^a^ grade (WD vs. MD + PD)	3.659	1.641–8.162	0.002*	3.316	1.462–7.524	0.004*
Lymph Node (Neg. vs. Pos.)	3.427	1.812–6.483	< 0.0001*	2.769	1.453–5.277	0.002*
Menopause (Pre vs. Post)	2.184	0.979–.4.870	0.056	**–**	**–**	**–**
ER^b^ (Neg. vs. Pos.)	0.843	0.476–1.491	0.557	**–**	**–**	**–**
PR^c^ (Neg. vs. Pos.)	0.717	0.380–1.356	0.306	**–**	**–**	**–**
Her-2^d^ (Neg. vs. Pos.)	1.784	0.999–3.184	0.050	**–**	**–**	**–**
TNBC^e^ (No vs. Yes)	0.839	0.450–1.563	0.579	**–**	**–**	**–**
Methylation (Meth. vs. Unmeth.)	2.892	1.528–5.474	0.001*	2.286	1.190–4.389	0.013*

^a^ *WD* Well differentiated, *MD* Moderately differentiated, *PD* Poorly differentiated

^**b**^ Estrogen receptor

^**c**^ Progesterone receptor

^**d**^ human epidermal growth factor receptor 2

^**e**^ Triple Negative breast cancer, *Significant Correlation (*P* < 0.05)

## Discussion

The expression of *Parkin* gene is frequently downregulated in a wide spectrum of tumors and cancer cell lines [4], while exogenous expression of *Parkin* protein inhibits cell proliferation and tumor growth in breast cancer [12]. Furthermore, the break at the *Parkin* gene/ FRA6E site have been linked with poor overall survival in breast carcinoma [28]. However, the molecular mechanism by which *Parkin* expression is down-regulated in tumors remains unclear. This was an important breakthrough for further investigations in the regulatory mechanism of *Parkin* gene expression and its pivotal contribution in the prognosis of breast cancer.

Loss of *Parkin* expression in a cell could persuade growth-promoting effect as a result of the failure of pro-apoptotic and cell cycle-suppressive regulations [29–31]. Our study indicated that in breast cancer, expression of *Parkin* was significantly lower/absent which confirms the results of earlier studies [4, 7–10, 28, 32]. Interestingly, unlike the previous study [28], we found a significant correlation of *Parkin* loss with the poorly/moderately differentiated grade of breast cancer, which highlights its relevance to breast carcinoma. We also found a statistically significant link between *Parkin* down-regulation and increased lymph node metastasis. This link suggests that *Parkin* gene has a metastasis suppressive role that is essential to prevent malignant progression in breast cancer as proposed by an earlier study in case of pancreatic cancer [11]. A previous report demonstrated no link between the *Parkin* expression and breast cancer subtypes [19]. In our study, we, however, found that the absence of *Parkin* expression appears to have a more profound significance in TNBC (Triple-negative breast cancer) cases which was also supported by the TCGA data. Another study has reported a short metastasis-free survival of patients with *Parkin* break but not with the loss of *Parkin* expression [28]. Our study, in contrast, demonstrates that a low *Parkin* expression is linked with a worse prognosis of breast cancer. Thus, in the present study reduced expression of *Parkin* and its correlation with an aggressive subtype like TNBC suggests a tumor-suppressive function of *Parkin.*

Reports have demonstrated somatic mutations and intragenic deletions of *Parkin* in colon cancer, glioblastoma, in addition to lung cancer [5]. Remarkably, *Parkin* mutations sometimes occur in the same domains or even at the same amino acids in cancer, which, when found in germline, causes neurodegeneration [5]. Here, we reported two novel somatic mutations/variants of *Parkin*; *Q34R* (exon 2) and *S167 N* (exon 4) which were absent in normal breast tissues. Interestingly, the mutation *Q34R* li*e*s in the UBL (ubiquitin-like region) domain that is involved in substrate ubiquitination [33, 34]. The variant *S167 N* was found to be situated in the RING0 domain, that plays a crucial role in *Parkin* inactivation [35]. The mutation *Q34R* has been reported yet, neither in cancer nor in PD while the status of *S167 N* variant does not have any known significance in case of Parkinson’s disease [36, 37]. Hence, it is possible to hypothesize that variant *S167 N* in Parkinson’s disease could be included in those genotypes which may influence the occurrence of *Parkin* somatic mutations at the same residue in cancer as indicated by an earlier study [5]. Notably, all mutated cases coincided with the loss of *Parkin* protein, however, we failed to get any significant association of these mutations. This observation indicates that *Parkin* mutation is a rare and not a predetermining factor for breast cancer as reported by an earlier study [38].

Addressing the molecular cause for *Parkin* loss, next we focused on its promoter methylation. It is reported that the *Parkin* promoter sequence contains distinct CpG islands that are frequently methylated in other cancers [15, 22]. As far as the authors are aware, we provide the first evidence that promoter methylation is a major event for the *Parkin* inactivation in breast cancer. To validate our hypothesis that *Parkin* is epigenetically regulated, we have further shown that the treatment of 5′-azacytidine, a methyltransferase inhibitor restores *Parkin* mRNA expression in different breast cancer cell lines. In this context, methylation at the *Parkin* promoter should not be viewed as a surrogate biomarker for the loss of *Parkin* but as a frequently identifiable attribute of breast cancer. Another substantial finding of the present study is that both *Parkin* promoter methylation and its expressional loss are associated with the advanced histological grade in breast cancer. Thus indicating towards a predominant link of methylation-mediated *Parkin* loss with poor pathophenotype in Indian breast cancer patients. We also observed that *Parkin* methylation but not its expression is linked with Her-2 positivity. The observed disparity in correlations provides a hint that *Parkin* methylation might be an early event with more therapeutic options whereas; *Parkin* loss may involve some other mechanisms besides the methylation, like transcriptional regulation. The clinical significance of molecular markers and their correlation with survival may carry a more favorable prognosis and increased chemotherapy sensitivity. Remarkably, in the present study, multivariate analysis indicated that the *Parkin* expression and methylation both are independent prognostic molecular markers of OS in breast cancer patients. Our results also illustrated that breast cancer patients with *Parkin* methylation exhibited poor overall survival while those with *Parkin* positive expression having a better 5-year patient survival.

## Conclusions

In conclusion, we demonstrated that the breast cancer patients have a hugely varied course for the regulation of *Parkin* expression, where promoter methylation seems to play a major role while *Parkin* mutation otherwise may have a somewhat little contribution. The low frequency of two mutations in the studied samples indicates that these might be passenger mutations although their damaging effect cannot be overruled. This study also speculates that *Parkin* methylation may have a negative prognostic effect on breast cancer. Moreover, our results also strongly support the notion that *Parkin* expression is a potential prognostic factor for advanced and triple-negative breast cancer and can be used as a biomarker. However, investigation of *Parkin* status with more attention towards the molecular subtypes like TNBC, which has a relatively worse prognosis and currently lacks sufficient attention, is warranted.

## Additional files

Additional file 1: **Table S1** Clinicopathological characteristics of breast cancer patients included for the study (*N* = 156). **Table S2** Representative table of primer sequence for PCR and their respective temperature 558 and amplicon size. **Table S3** Summary of mutations obtained along with codon and nucleotide alteration in breast tumor samples. (DOCX 24 kb)

Additional file 2: **Figure S1** PARK2 alterations. **Figure S2** PARK2 mutations. **Figure S3** PARK2 mutations in COSMIC. **Figure S4** A. Oncomine analysis showed loss of *Parkin* expression in different types of breast carcinoma cell line in comparison to other cancer cell lines using compendia cell lines data (61 cell lines) (https://www.oncomine.org/). (DOCX 925 kb)

## Data Availability

It is confirmed that all the data generated or analyzed during this study are included in this published article.

## Abbreviations

5′-azadC: 5-Aza-2-deoxycytidine
COSMIC: Catalogue of Somatic Mutations in Cancer analysis
DAB: 3, 3′-Diaminobenzidine
ER: Estrogen receptor
HER-2: Human epidermal growth factor receptor 2
IHC: Immunohistochemistry
MSP: Methylation-specific polymerase chain reaction
OS: Overall Survival
PD: Parkinson’s disease
PR: Progesterone receptor
Q34R: Glu34Arg
RT-PCR: Reverse transcriptase PCR
S167N: Ser167Asp
SSCP: Single-stranded conformational polymorphism
TCGA: The Cancer Genome Atlas
TNBC: Triple-negative breast cancer
UBL: Ubiquitin-like region
