# Supplementary material for: Parkin gene mutations are not common, but its epigenetic inactivation is a frequent event and predicts poor survival in advanced breast cancer patients
Source: BMC Cancer. 2019 Aug 20;19:820. doi: 10.1186/s12885-019-6013-6 (PMC6700819; doi:10.1186/s12885-019-6013-6)
Supplement: Supplementary file 1 — Table S1 Clinicopathological characteristics of breast cancer patients included for the study (N = 156). Table S2 Representative table of primer sequence for PCR and their respective temperature 558 and amplicon size. Table S3 Summary of mutations obtained along with codon and nucleotide alteration in breast tumor samples. (DOCX 24 kb) [file 12885_2019_6013_MOESM1_ESM.docx]

**Table S1: Clinicopathological characteristics of breast cancer patients included for the study (N=156)**

| S.No. | Variables | | Total (%) |
| --- | --- | --- | --- |
|  |  | |  |
| 1 | **Age**  **(Years)** | <50 | 30(19) |
|  |  | ≥50 | 126(81) |
| 2 | **Weight**  **(Kg)** | <60 | 75(48) |
|  |  | ≥60 | 81(52) |
| 3 | **Tumour size**  **(cm)** | <4` | 73(47) |
|  |  | ≥4 | 83(53) |
| 4 | **Clinical Stage** | I | 36(23) |
|  |  | II | 52(33) |
|  |  | III | 56(36) |
|  |  | IV | 12(8) |
| 5 | **Histological group^[[1]](#footnote-1)^** | WD | 65(42) |
|  |  | MD+PD | 91(58) |
| 6 | **Lymph Node** | Negative | 85(54) |
|  |  | Positive | 71(46) |
| 7 | **Menarche** | <15 | 42(27) |
|  |  | ≥15 | 114(73) |
| 8 | **Menopause** | Pre | 38(24) |
|  |  | Post | 118(76) |
| 9 | **Having Child** | No | 23(15) |
|  |  | Yes | 133(85) |
| 10 | **Abortion** | No | 104(67) |
|  |  | Yes | 52(33) |
| 11 | **Breast feeding** | No | 60(38) |
|  |  | Yes | 96(62) |
| 12 | **Use of contraceptive** | No | 105(67) |
|  |  | Yes | 51(33) |
| 13 | **ER** | Negative | 74(47) |
|  |  | Positive | 82(53) |
| 14 | **PR** | Negative | 103(66) |
|  |  | Positive | 53(34) |
| 15 | **Her-2** | Negative | 111(71) |
|  |  | Positive | 45(29) |
| 16 | **TNBC^[[2]](#footnote-2)^** | No | 106(68) |
|  |  | Yes | 50(32) |

**Table S2:** Representative table of primer sequence for PCR and their respective temperature 558 and amplicon size

| **Exon** | **Primer Sequence** | **Annealing Temp.(ᵒC)** | **Amplicon size (bp)** |
| --- | --- | --- | --- |
| 1 | F-5' GCCCCGTCATTGACAGTT 3' | 58 | 227 |
|  | R-5' GAGGCCTGGAGGATTTAACC 3' |  |  |
| 2 | 5' TAAGGGCTTCGAGTGATGCT 3' | 56 | 273 |
|  | R-5' GCATGAGCAATGGAGCTG 3' |  |  |
| 3 | F-5' CTCGCATTTCATGTTTGACA 3' | 58 | 394 |
|  | R-5' TAAATATGCACCCGGTGAGG 3' |  |  |
| 4 | F-5' GAGTTTCTTGTCTCAATTTAGATGC 3' | 63 | 290 |
|  | R-5' TTTCTTTTCAAAGACGGGTGA 3' |  |  |
| 5 | F-5' GTGGAAACATGTCTTAAGGAGTACA 3' | 64 | 225 |
|  | R-5' TTCCTGGCAAACAGTGAAGA 3' |  |  |
| 6 | F-5' GTCCAAAGAGATTGTTTACTGTGG 3' | 63 | 278 |
|  | R-5' GGGGGAGTGATGCTATTTTT 3' |  |  |
| 7 | F-5' GCCTTTCCACACTGACAGGTA 3' | 64 | 296 |
|  | R-5' AAATTCTTCTGCTAGGGTTTACG 3' |  |  |
| 8 | F-5' GGCAACACTGGCAGTTGATA 3' | 60 | 230 |
|  | R-5' GGAGCCCAAACTGTCTCATT 3' |  |  |
| 9 | F-5' AAGCAAGAAATCCCATGCAC 3' | 57 | 299 |
|  | R-5' TGTGCAAAAGCAAACAAGGA 3' |  |  |
| 10 | F-5' GGAACTCTCCATGACCTCCA 3' | 59 | 223 |
|  | R-5' GGAACTCTCCATGACCTCCA 3' |  |  |
| 11 | F-5' CCGACGTACAGGGAACATAAA 3' | 61 | 253 |
|  | R-5' ATGATTCTCCCCCAAAGAGC 3' |  |  |
| 12 | 12 F-5' GTTTGGGAATGCGTGTTTT 3' | 58 | 255 |
|  | R-5' AGAATTAGAAAATGAAGGTAGACA 3' |  |  |
|  |  |  |  |
| **Promoter** | **Primer Sequence** | **Annealing Temp.(ᵒC)** | **Amplicon size (bp)** |
| Methylated | F-5’ AGGTAAGTTTTTCGGTTGTTAAGC 3’ | 59.8 | 163 |
|  | R-5’ CTAAAAATCGTAATTCTAACGCGTA 3’ |  |  |
| Unmethylated | F-5’ GGTAAGTTTTTTGGTTGTTAAGTGA 3’ | 63 | 163 |
|  | R-5’ ACTAAAAATCATAATTCTAACACATA 3’ |  |  |
|  |  |  |  |
| **Gene** | **mRNA Primer Sequence** | **Annealing Temp.(ᵒC)** | **Amplicon size (bp)** |
| Parkin mRNA | 5’-AGAGCTCCATCACTTCAGGATT-3’ | 58˚C | 230 bp |
|  | 5’-CCCCTTCATGGTACGCTTCT-3’ |  |  |

**Table S3: Summary of mutations obtained along with codon and nucleotide alteration in breast tumor samples**

| S.No. | Tumor Stage | Histological grade^[[3]](#footnote-3)^ | | Lymph node | | Nucleotide alteration | | Amino acid alteration | | PARK-2 methylation | | PARK-2 expression^[[4]](#footnote-4)^ | |
| --- | --- | --- | --- | --- | --- | --- | --- | --- | --- | --- | --- | --- | --- |
| 1 | III | PD | Present | | 234-A→G | | **Glu**34**Arg** | | Absent | | Absent | |  |
| 2 | II | MD | Absent | | 234-A→G | | **Glu**34**Arg** | | Present | | Absent | |  |
| 3 | III | MD | Present | | 234-A→G | | **Glu**34**Arg** | | Present | | Absent | |  |
| 4  5  6 | II  III  III | WD  MD  PD | Absent  Present  Present | | 234-A→G  234-A→G  234-A→G | | **Glu**34**Arg**  **Glu**34**Arg**  **Glu**34**Arg** | | Present  Absent  Present | | Absent  Absent  Absent | |  |
| 7 | IV | PD | Absent | | 631-G→A | | **Ser**167**Asp** | | Absent | | Absent | |  |
| 8 | II | MD | Absent | | 631-G→A | | **Ser**167**Asp** | | Absent | | Absent | |  |
| 9 | IV | PD | Present | | 631-G→A | | **Ser**167**Asp** | | Absent | | Absent | |  |

1. WD*:* Well differentiated, MD: Moderately differentiated, PD: Poorly differentiated [↑](#footnote-ref-1)
2. TNBC: Triple negative breast cancer [↑](#footnote-ref-2)
3. WD: Well differentiated, MD: Moderately differentiated, PD: Poorly differentiated [↑](#footnote-ref-3)
4. Protein expression through IHC (Immunohistochemistry) [↑](#footnote-ref-4)
