# Supplementary material for: Parkin gene mutations are not common, but its epigenetic inactivation is a frequent event and predicts poor survival in advanced breast cancer patients
Source: BMC Cancer. 2019 Aug 20;19:820. doi: 10.1186/s12885-019-6013-6 (PMC6700819; doi:10.1186/s12885-019-6013-6)
Supplement: Supplementary file 2 — Figure S1 PARK2 alterations. Figure S2 PARK2 mutations. Figure S3 PARK2 mutations in COSMIC. Figure S4 A. Oncomine analysis showed loss of Parkin expression in different types of breast carcinoma cell line in comparison to other cancer cell lines using compendia cell lines data (61 cell lines) (https://www.oncomine.org/). (DOCX 925 kb) [file 12885_2019_6013_MOESM2_ESM.docx]

**Figure S1: PARK2 alterations A.** Total 2.3% mutations of Parkin gene in 12 breast cancer studies. **B.** Frequency of Parkin alterations in different studies of breast cancer in TCGA (The Cancer Genomic Atlas). **C.** Parkin alteration in different types of breast cancer adapted from the cBioPortal for Cancer Genomics (available at: [www.cbioportal.org](http://www.cbioportal.org)).

**Figure. S2: PARK2 mutations A.** Different types of mutations found in Parkin gene across 12 breast cancer studies in TCGA adapted from the cBioPortal for Cancer Genomics (available at: [www.cbioportal.org](http://www.cbioportal.org)). **B.** Frequency and types of Parkin alterations found in different studies of breast cancer in COSMIC (<https://cancer.sanger.ac.uk/cosmic>).

**Figure S3: PARK2 mutations in COSMIC** Different types of mutations found in Parkin gene in COSMIC (<https://cancer.sanger.ac.uk/cosmic>). **A.** Showing 1-10 of 16 entries. **B.** Showing 11-16 of 16 entries.

**Figure S4: A.** Oncomine analysis showed loss of *Parkin* expression in different types of breast carcinoma cell line in comparison to other cancer cell lines using compendia cell lines data (61 cell lines) (<https://www.oncomine.org/>).
